# Emerging adults’ digital technology engagement and mental health during the COVID-19 pandemic

**DOI:** 10.3389/fpsyg.2022.1023514

**Published:** 2022-11-17

**Authors:** Gregory E. Chase, Morgan T. Brown, Michaeline Jensen

**Affiliations:** Department of Psychology, University of North Carolina at Greensboro, Greensboro, NC, United States

**Keywords:** digital technology, screen time, COVID-19, mental health, emerging adulthood

## Abstract

Within the past decade, parents, scientists, and policy makers have sought to understand how digital technology engagement may exacerbate or ameliorate young people’s mental health symptoms, a concern that has intensified amidst the COVID-19 pandemic. Previous research has been far from conclusive, and a lack of research consensus may stem in part from widely varying measurement strategies (including subjective and objective measurement) around digital technology engagement. In a cross-sectional study of 323 university students, the present study seeks to understand the ways in which youth engagement with digital technology – across subjective and objective measurements, weekday and weekend distinctions, and social and non-social uses – is associated with mental health (as measured by depression, loneliness, and multidimensional mood and anxiety). The present study also tested a differential susceptibility hypothesis to examine whether COVID-19 related social isolation might exacerbate the potential harms or helps of digital technology engagement. Results yielded few observed associations between digital technology engagement and mental health, with little evidence of detrimental effects of observed or perceived time spent on digital technology. Rather, those significant findings which did emerge underscore potential protections conferred by social connections with friends (both online and offline), and that the loneliest students may be the most likely to be reaching out for these types of connections. It is important that the field move beyond crude (largely self-reported) measures of screen time to instead understand how and to what effect youth are using digital technologies, especially during the social corridor of emerging adulthood.

## Introduction

Intersections between digital technology engagement and mental health are of great interest, with media attention and research on the topic skyrocketing within the last 14 years (Hancock et al., 2019; Reeves et al., 2020). These intersections are of even more importance within the past 2–3 years of the COVID-19 pandemic (Richtel, 2021; Shrier, 2021), during which we have seen spikes in both frequency of digital technology engagement (Cielo et al., 2021; Kerekes et al., 2021) and psychological distress (Chen et al., 2021; Czeisler et al., 2021). Digital technology engagement has been recognized as both a protective and risk factor for mental health during the COVID-19 pandemic (Okabe-Miyamoto and Lyubomirsky, 2021), with recent work uncovering associations between technology engagement and poorer mental wellbeing (Smith et al., 2020; Li et al., 2021), as well as shining light on positive ways in which digital technology provided a valuable lifeline from widespread lockdowns and social distancing into the social, educational, and occupational worlds beyond one’s front door (Beaunoyer et al., 2020; Juvonen et al., 2021). While popular media has loudly argued that digital technology engagement is widely harmful (e.g., Twenge, 2017), it is important to consider how digital technology can be used to maintain social connections and protect against the negative consequences of the pandemic (Marciano et al., 2022). Thus, the current study seeks to test ways that digital technology engagement may be facilitative of or deleterious to young adult’s mental health during the pandemic.

The research to date on digital technology’s potential impacts on mental health is somewhat fraught. Many argue and some research suggests that more time spent on screens is harming youths’ mental health and potentially to blame for historical increases in depression, anxiety, and suicide since the advent of the smartphone (Twenge et al., 2018a,b; Twenge, 2020). However, a sizable body of studies using various methods (Nesi and Prinstein, 2015; Nesi et al., 2017; Jensen et al., 2019; Coyne et al., 2020), including meta-analyses and reviews (Appel et al., 2020; Odgers and Jensen, 2020; Tang et al., 2021) conclude that digital technology engagement has small, negligible, or mixed effects on youth mental health. It is important for us to sort through this apparent disorder in the literature and identify where (if anywhere) sizable risks and protections for mental health may occur, in service of staunching rising rates of psychological distress, especially amidst the pandemic when both screen time and mental health problems have had pronounced increases. It is likely that at least some of the lack of clarity in the literature to date stems from widely varying measurement strategies around digital technology engagement (Scharkow, 2016). Kaye et al. (2020) note that current methodological shortcomings include poor conceptualization of what is considered “screen time” and the use of non-standardized, self-report measures that are often underestimated by heavy users and overestimated by light users. Here we highlight three dimensions of measurement difference that are of potential interest: (1) Objective versus subjective (or perceived) measurement of digital technology engagement, (2) Weekday versus weekend measurement, and (3) Moving beyond monolithic assessments of general “screen time” to more nuanced *reasons* and *activities* young people engage in digital technology.

It is first important to consider objective versus subjective measurement of digital technology engagement. Though there is strong evidence indicating that self-reported frequency or duration of digital technology engagement is not a particularly accurate gauge of actual use (Boase and Ling, 2013; Parry et al., 2021), many studies continue to rely solely on self-reported digital technology engagement when determining links with mental health (Shaw et al., 2020). Admittedly, objective measurement of digital technology engagement (e.g., through device logs, systematic screenshots, or downloaded social media or message content; Andrews et al., 2015; Kaye et al., 2020) is considerably more onerous than gathering self-reports. However, these objective methods provide a more accurate metric of the actual time and activities one is engaged in online that is less impacted by participant’s attitudes and beliefs toward smartphone and social media use (Ellis et al., 2019). Thus, objective measures provide a clearer lens through which to understand digital technology and mental health associations (Parry et al., 2021), which is integral to addressing methodological limitations presented by previous research (Kaye et al., 2020). In fact, those few studies using objective measures of digital media engagement have little evidence of sizable or robust associations with mental health (Rozgonjuk et al., 2018; Hodes and Thomas, 2021; Sewall et al., 2022).

The timing of digital technology engagement, including the day of the week, appears to also be an important consideration. In a seminal article by Przybylski and Weinstein (2017) using cross-sectional data from a representative sample of English adolescents, results indicated that the distinction between moderate and potentially harmful screen time was considerably higher and less variable on the weekend, suggesting that the potential negative consequences of screen time are at a lower threshold during the weekday than on weekends. This is consistent with other studies in China and the United Kingdom (Khouja et al., 2019; Liu et al., 2019) which together suggest that the *day of the week* that adolescents engage in screen time may be an important distinction when considering harms of digital technology, perhaps because weekday screen time may displace other enriching activities that are beneficial to development in a way that weekend screen time does not.

Lastly and most importantly, it is important to understand whether different *types* of digital technology engagement are differentially linked with positive mental health flourishing or negative mental health functioning. For instance, theories of social support and connection (Cole et al., 2017) would suggest that engagement with social media that facilitate authentic social connection and supportive interactions would likely benefit youth mental health and wellbeing, and indeed research does suggest that social screen time (especially one-on-one interactions, including amidst the pandemic) seems to be associated with fewer mental health symptoms (Fumagalli et al., 2021; Marciano et al., 2022). It is also likely that uses of digital technology for targeted purposes like education, creativity, and mastery could also be beneficial for mental health (Sanders et al., 2019; Granic et al., 2020). Conversely, passive types of use without a social component may be more consistently linked with poorer mental health, whether because they are displacing alternate enriching activities that one *could otherwise* be engaging in, or because they serve as an avoidance strategy for internalizing youth (Kraut et al., 1998; Kim et al., 2020), though displacement may be less common amidst the pandemic when there were few face-to-face activities to displace. Overall, it is increasingly clear that rigorous research on digital technology engagement and mental health *must* respond to calls for more objective and nuanced assessment of the uses and affordances of different types of media use (Ellis et al., 2019; Kaye et al., 2020).

Not all young people experience the harms or the helps of social media in the same ways (Valkenburg and Peter, 2013). For instance, Orben et al. (2022) recently identified a window of sensitivity to social media effects at age 19 for both males and females, suggesting that the transition from adolescence to emerging adulthood may be an important developmental period to examine what ways social media may be protective or detrimental to wellbeing. Indeed, emerging adulthood (the period of study here) is a period when friendships are critical to development (Barry et al., 2016) and during which technology may be used in particularly satisfying ways to interact with friends.

Another potential point of differential susceptibility is the extent to which a young person is already struggling in their offline life (George and Odgers, 2015; Underwood and Ehrenreich, 2017). Here we focus on a salient struggle of the COVID-19 pandemic: Social isolation. Digital connection can serve as an important tool for social interaction and maintaining real-life social networks online (Mcmillan and Morrison, 2006), which may be especially pertinent during the COVID-19 pandemic when social isolation has prevented in-person connection (Juvonen et al., 2021). Thus, it is possible that those emerging adults who were the most isolated had the most to gain from digitally mediated social connection (a “poor get richer” hypothesis). There is also concern, however, that those emerging adults who were already struggling with social isolation induced by COVID-19 related lockdowns may have been particularly susceptible to the negative impacts of digital technology engagement on anxiety, depression, and loneliness during an already challenging time (consistent with the “the poor get poorer” hypothesis; Kraut et al., 2002).

### The current study

Given the murky landscape of current research on the intersections between digital technology engagement and youth mental health (including amidst the pandemic, Marciano et al., 2022), the present study takes a comprehensive approach in seeking to understand the ways in which emerging adult engagement with digital technology—across subjective and objective measurement strategies, weekday and weekend distinctions, and social and non-social uses—is associated with mental health symptoms (indexed by multidimensional mood and anxiety, depression, and loneliness). Specifically, we test the hypothesis that specific types of digital technology engagement (i.e., overall screen time and passive screen time) may be associated with poorer emerging adult mental health whereas other types of digital technology engagement (i.e., digital media for social purposes (especially private/one-on-one communication) and creativity screen time) may be associated with better emerging adult mental health, with more robust associations hypothesized for subjective self-reports than objective measures. We further hypothesize that, if associations with objective measures do arise, they are more likely to be seen on weekdays than weekends. We hypothesize this given past research on differential levels of digital technology engagement and impacts across the week. Finally, we test a differential susceptibility hypothesis and examine whether COVID-19 related social isolation might exacerbate the potential harms of non-social digital activities (a “vulnerable reactive” interaction, consistent with the “poor get poorer” hypothesis) but might also amplify the potential benefits of social digital activities (a protective enhancing interaction, consistent with the “poor get richer” hypothesis).

## Materials and methods

### Participants and procedures

Participants for this study were recruited via the University’s Psychology Department subject pool at a public, minority-serving university in the Southeastern United States in the Fall of 2020. In this semester, the university offered classes online, in-person, or in hybrid formats due to the COVID-19 pandemic. On-campus activities were extremely restricted at the time, and students were able to live on-campus in a limited capacity (*n* = 177; 54.8% of our sample). Participants had to be at least 18 years of age to participate. Eligible participants were routed to an online Qualtrics survey where they provided informed consent before completing the survey assessment, for which they were awarded course credit. All procedures, protocols, and measures were approved by the university’s Institutional Review Board (approval #21-0139).

A total of 393 participants consented to the study, with 30 (7.6%) excluded from present analyses due to data quality issues (i.e., completing <20% of the full survey assessment, survey duration <1/5th of the average length of survey completion) and an additional 40 participants (10.2%) excluded due to not owning an iPhone (necessary for our objective screen time measurement). Of the resulting analytic sample (*N* = 323), most were first year university students (55.1%) and identified as female (74.6%). The sample was racially/ethnically diverse (43.7% White, not-Hispanic; 43.4% Black, not-Hispanic; 14.8% Hispanic/Latinx; 6.8% Asian, 2.8% American Indian or Alaskan Native, 1.2% Middle Eastern or North African, 0.9% Native Hawaiian, and 0.9% identified as another race or ethnicity).

### Measures

#### Demographics

Participants reported on key demographics at the beginning of the Qualtrics survey. Covariates included within the current study were participant *Age* in years (*M* = 19, *SD* = 2.13), and *Gender Identity* which was dummy coded into *Male Identifying* (21.2%), *Female Identifying* (74.6%), and *Other Identifying* (2.2%).

#### Perceived COVID-19 related social isolation

The 109-item Epidemic-Pandemic Impacts of Inventory Adolescent Adaptation (EPII-A; Morris et al., 2020) was administered to assess whether the participant perceived that the “COVID-19 pandemic has impacted you in the way described” on a binary *yes* (1) or *no* (0) scale (with an option for “does not apply” recoded here as 0). The item querying whether the participant was “Separated from friend(s)” was used as a dichotomous measure of perceived COVID-19 related social isolation, to which 56.3% responded “*yes*.”

#### Perceived time spent on technology

##### Perceived COVID-19 related increases in screen time

One item from the EPII-A (Morris et al., 2020) queried whether the participant had “Spent more time on screens and devices” (74.6% *yes*) amidst the COVID-19 pandemic.

##### Perceived social media and screen time

Participants were first asked to estimate the “average amount of time you spend on your phone daily” (*perceived screen time*) and “the average amount of time you spend on social media daily” (*perceived social media time*) in hours (range *0 = <1 h* to 23 *= 23–24 h*). Participants’ reports revealed an average of 6.46 h (*SD* = 2.89) of daily screen time, with an average of 4.29 h (*SD* = 2.24) spent on social media.

#### Perceived time spent online and offline for social connection

The Electronic Interaction Scale for Time (EIS_T; Nesi and Prinstein, 2015) queried the average amount of time participants use specific technologies to connect with their friends on a “typical day.” Participants indicated “on a typical day in the last month, how much time do you spend…” engaging with friends (on a 7-point scale ranging from *0 = I do not use this, 1 = 1 h or less, 6 = 9 or more hours*) for *face-to-face communication* (i.e., time they are talking for fun or social reasons, not just sitting together in class; *M* = 3.01, *SD* = 1.78), *phone calls, FaceTime, and Skype* (*M* = 2.61, *SD* = 1.61), *text messaging* (*M* = 3.07, *SD* = 1.72), *private social media* (e.g., Snapchat, private messaging on Facebook or emailing; *M* = 2.83, *SD* = 1.66), and *public social media* (e.g., Facebook, Instagram, Twitter; *M* = 2.04, *SD* = 1.62).

#### Objective screen time

Given the limitations of subjective self-reports on screen time (Scharkow, 2016), we piloted a procedure for coaching participants through the use of their native smartphone screen time tracking app to report (more) objective screen time. Similar methods for obtaining objective screen time measures have recently been used in adult (Ohme et al., 2021) and emerging adult (Hodes and Thomas, 2021) samples. iPhone users (*N* = 323) were instructed step by step on how to access and use the native iPhone Screen Time app (with the aid of screenshots embedded within the Qualtrics survey showing where to click) to find the amount of time (in hours and minutes) spent on their phone for different uses: (1) *Social* (e.g., Instagram, Snapchat, Facebook, TikTok); (2) *Creativity* (e.g., Camera, Photos); (3) *Entertainment* (e.g., Netflix, YouTube, Spotify); (4) *Education* (e.g., Canvas learning management system); and (5) *Productivity* (e.g., notes, mail, calendar). Android users (*n* = 40) completed a similar procedure but given that the categories reported on by the two platforms’ native screen time tracking apps differ, we focus here on the majority of the sample (88.98%) who were iPhone users. Screen time categories are determined by Apple’s Screen Time application and vary slightly from participant to participant based on what applications they have downloaded on their phone and use. Given past studies which have highlighted differences in the quantity and impacts of weekend versus weekday screen time (Przybylski and Weinstein, 2017), we asked participants to report each of these amounts separately for the previous Wednesday (to capture *weekday screen time*) and the previous Saturday (to capture *weekend screen time*) prior to survey administration. Participants reported on objective measures of screen time after the perceived measures of screen time and social media variables so that the objective measures did not bias their perception of time spent online. Descriptive statistics of objective measures of screen time can be found in Table 1.

**Table 1:** Means and standard deviations of objective time spent on technology in hours.

Objective screen time (in hours)	*M*	*SD*	Min	Max
Social weekday	3.47	2.55	0.00	22.32
Creativity weekday	1.07	1.76	0.00	11.52
Entertainment weekday	1.62	2.10	0.00	12.83
Education weekday	0.54	1.27	0.00	15.00
Productivity weekday	0.38	0.69	0.00	4.55
Social weekend	3.42	2.60	0.00	17.55
Creativity weekend	0.98	1.69	0.00	12.75
Entertainment weekend	1.66	2.35	0.00	18.90
Education weekend	0.41	1.32	0.00	15.00
Productivity weekend	0.29	0.72	0.00	5.90

*N* = 323.

#### Mental health

##### Depressive symptoms

Participants reported on their past month depressive symptoms using the well-validated 13-item Short Mood and Feelings Questionnaire (SMFQ; Angold et al., 1995), in which participants are provided I-statements that describe depressive moods and behaviors (e.g., “I felt miserable or unhappy”) on a 3-point Likert scale ranging from *0 = Not True to 2 = True*. The original timeframe for responding was adapted from *past two weeks* to *past month* to align with other study measures. A mean score of the 13 items was created (*M* = 0.65, *SD* = 0.54), which evidenced strong internal consistency in this sample (*α* = 0.93).

##### Multidimensional mood and anxiety

Anxiety symptoms were assessed using the well-validated Mini Mood and Anxiety Symptom Questionnaire (Mini-MASQ; Casillas and Clark, 2000) which is composed of mean scores on three subscales of mood symptoms: Anhedonic depression (8-items; e.g., “Felt like nothing was very enjoyable;” *M* = 2.40, *SD* = 0.63; *α* = 0.81), anxious arousal (10-items; e.g., “Hands were cold or sweaty;” *M* = 0.23, *SD* = 0.41; *α* = 0.85), and general distress (8-items; e.g., “Felt tense or high strung;” *M* = 0.46, *SD* = 0.65; *α* = 0.91). Participants were instructed to indicate the extent to which they experience each symptom on a 5-point Likert scale ranging from *0 = not at all to 4 = extremely*. The original timeframe of *past week* was adapted to tap *past month* symptoms in accordance with other study measures.

##### Loneliness

Loneliness was assessed using the UCLA Loneliness Scale, version 3 (Russell, 1996). This scale averages 20 items that measure subjective feelings of loneliness and social isolation (e.g., “I feel completely alone”; *M* = 1.88, *SD* = 0.76; *α* = 0.96) using a 4-point Likert scale ranging from *0 = I never feel this way to 3 = I often feel this way*. The original timeframe of *lifetime* loneliness was adapted for *past month* loneliness.

### Data analytic plan

All analyses were conducted in Mplus 8.7 (Muthén and Muthén, 1998–2021) with the help of Hallquist and Wiley’s (2018) Mplus automation package. All models used a maximum likelihood estimator with robust standard errors (MLR) and full information maximum likelihood (FIML) for missing data handling (Enders and Bandalos, 2001). We regressed each indicator of mental health (depression, anhedonic depression, anxious arousal, general distress, and loneliness) on each indicator of technology engagement alongside age and gender as covariates. We also tested whether each of these associations between indicators of technology engagement and mental health were moderated by perceived COVID-19 social isolation. Indicators included in interaction terms were mean centered to facilitate interpretation (Aiken et al., 1991) and significant interactions were probed among the socially isolated and non-socially isolated participants. Given the many comparisons (180 total models) inherent in testing these many hypothesized associations, we used the Benjamini Hochberg False Discovery Rate (FDR) procedure to adjust significant tests for multiple comparisons (Benjamini and Hochberg, 1995). For transparency, we report traditional *p*-values in all tables, with those that meet FDR-corrected significance levels marked with an asterisk.

## Results

### Direct associations between digital technology engagement and mental health

Results from tests of associations between digital technology engagement and mental health can be found in Table 2.

**Table 2:** Direct associations between digital technology engagement and mental health.

	Past month mental health									
	Anhedonic depression		Anxious arousal		General distress		Depressive symptoms		Loneliness symptoms	
	*b* (*SE*)	*p*	*b* (*SE*)	*p*	*b* (*SE*)	*p*	*b* (*SE*)	*p*	*b* (*SE*)	*p*
Objective time spent on technology										
Weekday										
Creativity	−0.03 (0.02)	0.160	0.01 (0.02)	0.724	−0.02 (0.02)	0.484	0.01 (0.02)	0.734	−0.02 (0.02)	0.283
Educational	**−0.07 (0.02)**	**0.005**	<0.01 (0.02)	0.877	−0.02 (0.02)	0.439	−0.04 (0.02)	0.061	<0.01 (0.03)	0.988
Entertainment	0.02 (0.02)	0.244	0.02 (0.01)	0.179	0.03 (0.02)	0.126	0.02 (0.02)	0.236	−0.04 (0.02)	0.122
Productivity	<0.01 (0.05)	0.991	0.01 (0.03)	0.772	−0.04 (0.04)	0.377	−0.02 (0.04)	0.678	−0.01 (0.06)	0.927
Social	0.02 (0.01)	0.184	0.01 (0.01)	0.152	0.02 (0.02)	0.250	0.01 (0.01)	0.301	<0.01 (0.02)	0.845
Weekend										
Creativity	−0.03 (0.02)	0.219	0.01 (0.02)	0.446	−0.01 (0.02)	0.655	0.02 (0.02)	0.480	−0.02 (0.03)	0.532
Educational	−0.03 (0.03)	0.244	0.01 (0.02)	0.471	>−0.01 (0.02)	0.850	−0.03 (0.02)	0.109	−0.06 (0.03)	0.055
Entertainment	0.01 (0.02)	0.609	0.02 (0.01)	0.189	0.01 (0.02)	0.469	0.01 (0.02)	0.373	−0.01 (0.02)	0.749
Productivity	−0.02 (0.05)	0.746	0.05 (0.05)	0.314	−0.01 (0.05)	0.811	−0.03 (0.04)	0.502	−0.10 (0.06)	0.110
Social	0.02 (0.01)	0.092	0.01 (0.01)	0.369	0.01 (0.02)	0.427	0.02 (0.01)	0.124	0.01 (0.02)	0.476
Perceived time spent on technology										
Screen time	<0.01 (0.01)	0.757	<0.01 (0.01)	0.738	0.01 (0.01)	0.598	0.02 (0.01)	0.100	<0.01 (0.02)	0.973
Social media time	<0.01 (0.02)	0.903	>−0.01 (0.01)	0.749	>−0.01 (0.02)	0.785	0.01 (0.02)	0.423	0.01 (0.02)	0.617
Perceived COVID-19 screen time impact	−0.03 (0.08)	0.691	**0.09 (0.04)**	**0.043**	0.06 (0.08)	0.432	0.08 (0.06)	0.194	0.05 (0.10)	0.628
Perceived time spent online and offline for social connection										
Face-to-face	**−0.11 (0.02)**	<0.001*	−0.01 (0.01)	0.717	**−0.06 (0.02)**	**0.003**	−0.03 (0.02)	0.137	**0.10 (0.02)**	<0.001*
Phone calls	−0.04 (0.02)	0.085	>−0.01 (0.02)	0.827	−0.03 (0.02)	0.250	−0.02 (0.02)	0.464	**0.06 (0.03)**	**0.049**
Texting	**−0.05 (0.02)**	**0.033**	>−0.01 (0.02)	0.830	−0.02 (0.02)	0.187	−0.02 (0.02)	0.319	**0.08 (0.03)**	0.002*
Private social media	**−0.06 (0.02)**	**0.007**	−0.01 (0.02)	0.768	**−0.05 (0.02)**	**0.033**	−0.04 (0.02)	0.055	**0.10 (0.03)**	<0.001*
Public social media	−0.04 (0.02)	0.085	0.01 (0.02)	0.441	−0.02 (0.02)	0.511	−0.02 (0.02)	0.306	**0.06 (0.03)**	**0.032**

*N* = 323. Associations between each indicator of Digital Technology Engagement and each type of mental health symptom are tested in single level regressions, alongside covariates of age, and dummy coded gender. Raw regression coefficients (*b*), standard errors (SE), and *p*-values are reported. Significant (*p* ≤ 0.05) associations are bolded. Coefficients that met FDR-corrected significance levels are asterisked.

#### Objective time spent on technology

As seen in Table 2, students’ objective weekday and weekend reports of various screen time categories were mostly not related to past month mental health, with one exception: Higher weekday educational screen time was related to lower levels of anhedonic depression in the past month (*β* = −0.13), though this association did not meet FDR-corrected significance levels.

#### Perceived time spent on technology

Those students who reported perceiving more time on social media, more time on screens, and more COVID-19 related increases in screen time were mostly not significantly more or less likely to report experiencing mental health problems in the past month (Table 2), with one exception: Those students who perceived that the pandemic had caused their screen time to increase endorsed higher levels of anxious arousal in the past month (*β* = 0.09), though this association did not meet FDR-corrected significance levels.

#### Perceived time spent online and offline for social connection

Those students who perceived themselves as engaging in more face-to-face communication with friends during the pandemic endorsed lower levels of anhedonic depression (*β* = −0.30; met FDR-corrected significance levels) and general distress (*β* = −0.18; did not meet FDR-corrected significance levels). Students who used text messaging with their friends more also endorsed lower levels of anhedonic depression (*β* = −0.13), though this association did not meet FDR-corrected significance levels. Students who interacted with friends through private social media more endorsed fewer symptoms of anhedonic depression (*β* = −0.16) and general distress (*β* = −0.13), though neither of these associations met FDR-corrected significance levels. An interesting pattern emerged between students’ perceived frequency of online and offline social time and symptoms of loneliness where all indicators significantly predicted higher levels of student loneliness. Students who talked more with friends via face-to-face (*β* = 0.22), text messaging (*β* = 0.18), and private social media (*β* = 0.23) methods all endorsed greater levels of loneliness, which met FDR-corrected significance levels and students who perceived themselves as using more phone calls, FaceTime, and Skype (*β* = 0.12) and public social media (*β* = 0.12) to connect with friends also endorsed higher levels of loneliness, though results did not meet FDR-corrected significance levels.

### Differential associations for more isolated students: Associations between digital technology engagement and mental health as moderated by COVID-19-related social isolation

Students who reported experiencing COVID-19 related social isolation tended to report more symptoms of depression (*b* = 0.14, *SE* = 0.06, *p* = 0.023; *β* = 0.13) and general distress (*b* = 0.15, *SE* = 0.08, *p* = 0.047; *β* = 0.11) though both did not meet FDR-corrected significance levels. Results from tests of interactions modeling potential differential susceptibility can be found in Table 3.

**Table 3:** Differential associations for more isolated students: associations between digital technology engagement and mental health as moderated by COVID-19-related social isolation.

	Past month mental health									
	Anhedonic depression		Anxious arousal		General distress		Depressive symptoms		Loneliness symptoms	
	*b* (*SE*)	*p*	*b* (*SE*)	*p*	*b* (*SE*)	*p*	*b* (*SE*)	*p*	*b* (*SE*)	*p*
Objective Time Spent on Technology										
Weekday										
Creativity × Isolation	−0.02 (0.04)	0.648	<0.01 (0.04)	0.934	−0.02 (0.04)	0.585	0.01 (0.04)	0.860	0.06 (0.04)	0.155
Educational × Isolation	−0.02 (0.05)	0.733	−0.05 (0.04)	0.236	−0.02 (0.05)	0.635	0.01 (0.05)	0.896	0.08 (0.08)	0.311
Entertainment × Isolation	**0.07 (0.03)**	**0.026**	<0.01 (0.03)	0.920	0.05 (0.04)	0.139	0.06 (0.03)	0.053	−0.03 (0.04)	0.520
Productivity × Isolation	−0.10 (0.11)	0.339	−0.05 (0.06)	0.376	−0.09 (0.08)	0.268	−0.08 (0.08)	0.303	0.17 (0.13)	0.179
Social × Isolation	−0.02 (0.03)	0.322	0.03 (0.02)	0.110	0.02 (0.03)	0.524	0.03 (0.02)	0.195	−0.01 (0.03)	0.820
Weekend										
Creativity × Isolation	−0.02 (0.04)	0.640	0.01 (0.03)	0.782	> − 0.01 (0.04)	0.970	0.05 (0.04)	0.148	0.03 (0.06)	0.609
Educational × Isolation	−0.05 (0.05)	0.381	−0.03 (0.03)	0.374	−0.02 (0.05)	0.703	> − 0.01(0.04)	0.109	**0.12 (0.05)**	**0.019**
Entertainment × Isolation	0.05 (0.03)	0.126	−0.01 (0.03)	0.734	0.02 (0.03)	0.636	0.04 (0.03)	0.179	0.07 (0.04)	0.121
Productivity × Isolation	<0.01 (0.11)	0.996	**−0.19 (0.07)**	**0.009**	**−0.19 (0.08)**	**0.020**	−0.10 (0.08)	0.184	0.17 (0.12)	0.154
Social × Isolation	−0.03 (0.03)	0.401	0.01 (0.02)	0.560	0.02 (0.03)	0.549	0.03 (0.03)	0.266	0.05 (0.04)	0.173
Perceived Time Spent on Technology										
Screen time × Isolation	0.03 (0.03)	0.197	>−0.01 (0.02)	0.895	−0.03 (0.03)	0.323	<0.01 (0.02)	0.948	0.01 (0.03)	0.665
Social media time × Isolation	0.03 (0.03)	0.319	0.02 (0.02)	0.407	0.02 (0.03)	0.589	0.05 (0.03)	0.065	0.03 (0.04)	0.445
Perceived COVID-19 screen time impact × Isolation	0.03 (0.15)	0.861	−0.09 (0.09)	0.326	−0.03 (0.15)	0.837	0.02 (0.13)	0.876	0.16 (0.21)	0.440
Perceived time spent online and offline for social connection										
Face-to-face × Isolation	−0.04 (0.04)	0.338	>0.01 (0.03)	0.964	−0.01 (0.04)	0.833	0.01 (0.04)	0.748	−0.04 (0.05)	0.383
Phone calls × Isolation	0.01 (0.05)	0.891	−0.04 (0.03)	0.194	**−0.10 (0.05)**	**0.026**	−0.05 (0.04)	0.194	0.05 (0.06)	0.363
Texting × Isolation	<0.01 (0.04)	1.000	−0.03 (0.03)	0.351	**−0.10 (0.05)**	**0.031**	−0.03 (0.04)	0.489	0.09 (0.06)	0.116
Private social media × Isolation	−0.03 (0.05)	0.549	−0.02 (0.03)	0.505	**−0.11 (0.05)**	**0.020**	−0.01 (0.04)	0.781	0.07 (0.05)	0.207
Public social media × Isolation	−0.04 (0.05)	0.416	>0.01 (0.04)	0.999	−0.08 (0.05)	0.103	>0.01 (0.04)	0.999	0.07 (0.05)	0.176

*N* = 323. Associations between each indicator of Digital Technology Engagement, COVID-19 related social isolation, and their interaction with each type of mental health symptom are tested in single level regressions, alongside covariates of age and dummy coded gender. Raw regression coefficients (*b*), standard errors (SE), and *p*-values for interaction terms only are reported here, with simple slopes for significant interactions reported in the Results section. Significant (*p* ≤ 0.05) associations are bolded. No interaction terms met FDR-corrected significance levels.

#### Objective time spent on technology

As seen in Table 3, there were four significant interactions between measures of objective screen time and COVID-19 related social isolation, though none of these met FDR-corrected significance levels. Specifically, probing significant interactions between COVID-19 related social isolation and weekend productivity screen time revealed that those students who had experienced COVID-19 related social isolation saw significant associations between weekend productivity screen time and less anxious arousal (*b* = −0.06, *SE* = 0.02, *p* = 0.023; *β* = −0.10) and less general distress (*b* = −0.12, *SE* = 0.05, *p* = 0.032; *β* = −0.13), whereas those who had not experienced COVID-19 related social isolation saw non-significant associations between weekend productivity screen time and anxious arousal (*b* = 0.13, *SE* = 0.07, *p* = 0.052; *β* = 0.23) and general distress (*b* = 0.07, *SE* = 0.06, *p* = 0.259; *β* = 0.08). Socially isolated students saw a significant association between weekday entertainment screen time and more anhedonic depression (*b* = 0.06, *SE* = 0.03, *p* = 0.022; *β* = 0.19) whereas those students who were not socially isolated amidst the pandemic saw non-significant associations between weekday entertainment screen time and less anhedonic depression (*b* = −0.02, *SE* = 0.02, *p* = 0.467; *β* = −0.05). Students who had not experienced COVID-19 related social isolation saw significant associations between weekend educational screen time and less loneliness (*b* = −0.11, *SE* = 0.04, *p* = 0.003; *β* = −0.19) whereas socially isolated students saw a non-significant association between weekend educational screen time and loneliness (*b* = 0.01, *SE* = 0.04, *p* = 0.709; *β* = 0.02).

#### Perceived time spent on technology

No significant interactions between COVID-19 perceived social isolation and perceived social media time, screen time, or COVID-19 related increases in screen time emerged (as seen in Table 3).

#### Perceived time spent online and offline for social connection

There were three significant interactions between perceived frequency of online social time (Table 3; phone calls, texting, and private social media) and COVID-19 related social isolation, though none of these met FDR-corrected significance levels. Among those who were experiencing COVID-19 related social isolation, those who engaged more frequently with friends via phone calls (*b* = −0.07, *SE* = 0.03, *p* = 0.031; *β* = −0.18), texting (*b* = −0.07, *SE* = 0.03, *p* = 0.033; *β* = −0.18), and private social media (*b* = −0.09, *SE* = 0.03, *p* = 0.005; *β* = −0.24) reported less general distress, whereas those who had not experienced COVID-19 related social isolation saw non-significant associations between phone calls (*b* = 0.03, *SE* = 0.03, *p* = 0.319; *β* = 0.07), texting (*b* = 0.03, *SE* = 0.03, *p* = 0.332; *β* = 0.08), and private social media (*b* = 0.01, *SE* = 0.03, *p* = 0.652; *β* = 0.03) and general distress.

## Discussion

The COVID-19 pandemic saw unprecedented spikes in social isolation, mental health symptoms, and time spent on digital technologies; educators, practitioners, parents, and social scientists all worry about potential lasting consequences for young people’s wellbeing. The present study sought to understand the ways in which emerging adult engagement with digital technology (across subjective and objective measurement strategies, weekdays and weekends, and social and non-social uses) is associated with mental health amidst the COVID-19 pandemic, with results suggesting few robust harms imparted by digital technology engagement but potential benefits imparted when youth are making close social connections (both online and offline).

Overall, there was little evidence of detrimental associations of observed or perceived *time spent* on digital technology with mental health. Of 65 potential direct associations, only two emerged as significant: More weekday objective educational screen time was associated with less anhedonic depression and perceived COVID-19 screen time increases were associated with more anxious arousal. It is perhaps not surprising that those emerging adults who were experiencing more anxiety also tended to perceive their screen time as having increased – whether this be because they have been primed by the media and their parents to see screen time as harmful and perhaps to blame for their mental health symptoms (Kamenetz, 2021) or because of a more objective link between pandemic-screen time increases and anxiety (though the lack of robust associations across other more objective indicators would suggest not). Given that, amidst the pandemic in Fall 2020, much of college education was occurring on screens, it may be that the observed association between objectively-recorded education-related screen time and anhedonic depression is reflective of well-established links between the amotivation characteristic of anhedonia and low academic engagement (Fletcher et al., 2022); that is, those students who were experiencing anhedonia were probably the least likely to be signing on to their courses’ learning management systems to check homework or watch lecture videos. It must be noted, though, that neither of these associations stood up to corrections for multiple comparisons, suggesting that they are possibly spurious and should be interpreted only with caution.

In contrast to the largely null results around time spent on technology (whether assessed objectively versus subjectively on weekday or weekends), we saw slightly more robust findings around the perceived amount of time spent connecting socially with friends online (and face-to-face), where the more socially connected students tended to report less anhedonic depression and general distress (perhaps indicative of a protective association between online and offline social connection and better mental health) but more loneliness over the past month (though again, not all associations stood up to corrections for multiple comparisons). On the one hand, it seems somewhat counterintuitive that we would see different directions of association for these three indicators of mental health. However, perhaps not; it very well may be that higher levels of social connection cause emerging adults to feel less down and distressed (or, conversely, that those students who are the least down and distressed are also feeling the most amenable to fostering social connections with their peers). Also, those students who are experiencing the highest levels of loneliness (a very social form of internalizing symptom) are the most motivated to reach out to their peers for social connection. It is also notable that we seem to see more robust associations with indicators of one-on-one communication with a closer social network (i.e., face-to-face, text messaging, and private social media) relative to more public forms of social media connections (i.e., public social media). This underscores the idea that it might matter less whether young people are making their social connections in online or offline spaces, and matter more whether these social connections are meaningful and authentic (likely easier in private channels of communication).

In addition to the direct associations with social connections above, there was also some (limited) evidence for differential susceptibility for those who were experiencing the most COVID-19 related social isolation. Seven significant interactions revealed one significant “poor get poorer” interaction (socially isolated students saw an association between weekday objective entertainment screen time with more anhedonic depression), one “rich get richer” interaction (non-socially-isolated students saw an association between weekend educational screen time with less loneliness), and five “poor get richer” interactions (socially isolated students saw associations between more weekend objective productivity screen time and less anxious arousal and general distress alongside stronger associations between more perceived frequency of social connection with friends via phone/video call, text message, and private social media with less general distress). It is somewhat difficult to know what to make of this mixed bag of interactions, especially given that none of these seven interactions (of 90 possible) maintained statistical significance once multiple comparisons were accounted for and must thus be interpreted cautiously and in light of the fact that they may be unlikely to replicate.

Further, the significant associations observed here across all models were small by standard metrics (Bowman, 2012), accounting for a relatively small proportion of the overall variance in mental health symptoms. Thus, we must conclude that overall the present study yields little support for quantity of digital technology engagement as a risk factor for mental health, but some (limited) evidence for potential protections conferred by online and offline social connections against mental health symptoms, particularly for those who were feeling socially isolated by the COVID-19 pandemic. The COVID-19 pandemic is likely to have lasting impacts on youth’s mental health, and current research has started to uncover ways that youth may have difficulties adjusting to school, work, and social activities. Though the current study collected data in the Fall of 2020 amidst the height of the pandemic, it is likely that these experiences of depression, anxiety, and loneliness persist beyond the pandemic, and that the processes here will continue to be of importance. It is thus important that future researchers consider ways that social connection may be beneficial to youth mental health outside of the COVID-19 pandemic and explore different ways (beyond platform usage) that youth are able to connect with their peers online.

### Limitations

These findings must be interpreted in light of several specific study limitations,. First, as this study was our first foray into piloting the use of the Apple Screen Time app to track objective time spent on the students’ iPhone, some informative lessons were learned here. Notably, we excluded Android users from the present analysis, as the categories of Screen Time tracking on that platform differ from iOS. While Android users made up a small proportion of the current sample (11.05%), future research should consider cross-platform harmonization of screen time measures for a more representative sample that does not rely solely on iPhone using participants. Further, we became aware after data collection that Apple offers an option to “Share across devices” for screen time data (off by default), which counts time accrued on all devices (e.g., iPhone, iPad, and Mac computers) toward the tracked screen time available in the app. Thus, we cannot be certain here that all students here are being scored on the same metric/number of devices (i.e., some students may have higher objective screen time values because they spend lots of time on their MacBook whereas others may be spending lots of time on an iPhone specifically). Future research seeking to employ objective screen time should consider querying whether this setting turned on, and if so, what other devices the student has linked to their Apple ID that would be tracked and/or using recently developed protocols for uploading screen time screenshots that capture this setting (Sewall et al., 2022).

Second, as with all cross-sectional research, it is important to remember that the present study cannot be used for causal inference and does not tell us definitively what the drivers of the observed associations are. This concern is somewhat disputable, as few significant associations emerged to interpret (and those that were significant were fairly small), though those that did could be illustrating processes in which technology engagement drives mental health, in which mental health drives degree and type of engagement with technology, or indeed in which third variables (e.g., predispositions) drive both. It is imperative that future research employ longitudinal and experimental designs to clarify potential causal associations.

## Conclusion

The present study offers further support for the growing consensus that quantity of engagement with digital technology is not universally or robustly harmful, and that our quest to prevent young adult mental health problems would be best served by focusing on the specific ways in which young people use digital technologies to meet their social needs. The importance of social connection was especially true amidst the social isolation of the COVID-19 pandemic, but certainly extends beyond the pandemic, especially in the adolescent and emerging adult periods when social connections are so developmentally salient (Barry et al., 2016). This study is strengthened by a transparent approach to reporting findings across different possible operationalizations of digital technology engagement and mental health and by data collection in real-time amidst the COVID-19 pandemic. This study suggests few consistent mental health risks imparted by time spent online (regardless of measurement strategy, including a somewhat novel self-report strategy using Apple’s Screen Time app) and rather highlight some potential mental health benefits of connecting socially with friends online and offline. Nonetheless, we believe that it is important that researchers commit to carefully considering the impact of using subjective measures of digital technology engagement and to consider moving to objective measurement. This could take the form of objective measures of *quantity* of screen time (ideally separately for different types of uses) like device logs, usage screenshots, or the method employed here, or innovative methods to better understand the objective content of *what youth are doing online* like the Effortless Assessment of Risk States program (EARS; Lind et al., 2018) or the methods employed in the Human Screenome Project (Reeves et al., 2020). We will be best positioned to understand the role of digital technologies in mental health if we have a more accurate and rich understanding of what types of activities (e.g., social comparison, social connection, cyberbullying, information seeking, passive scrolling) youth are engaging in online.

Importantly, results suggest that the loneliest students are also the more likely to seek out online social connections. Those invested in the mental health of emerging adults should carefully consider the ways in which virtual tools may help foster social connections in this important developmental period, especially during crises during which face-to-face social connections are undermined (as was/is the case in the ongoing COVID-19 pandemic). Social scientists should also attend further to the importance of moving beyond crude measures of screen *time* to instead understand *how* youth are using digital technologies.

## Data availability statement

The raw data supporting the conclusions of this article will be made available by the authors, without undue reservation.

## Ethics statement

The studies involving human participants were reviewed and approved by University of North Carolina at Greensboro Institutional Review Board [#21-0139]. The patients/participants provided their written informed consent to participate in this study.

## Author contributions

GC, MB, and MJ contributed to conception and design of the study. MB organized the database. GC performed the statistical analyses and wrote the first draft of the manuscript. MB and MJ reviewed and edited the manuscript. All authors have substantially contributed to the manuscript, approved this submission, and agreed to be responsible for the scientific accuracy and integrity of this work.

## Conflict of interest

The authors declare that the research was conducted in the absence of any commercial or financial relationships that could be construed as a potential conflict of interest.

## Publisher’s note

All claims expressed in this article are solely those of the authors and do not necessarily represent those of their affiliated organizations, or those of the publisher, the editors and the reviewers. Any product that may be evaluated in this article, or claim that may be made by its manufacturer, is not guaranteed or endorsed by the publisher.
